# SIRT3-mediated deacetylation of NLRC4 promotes inflammasome activation

**DOI:** 10.7150/thno.55573

**Published:** 2021-02-15

**Authors:** Chenyang Guan, Xian Huang, Jinnan Yue, Hongrui Xiang, Samina Shaheen, Zhenyan Jiang, Yuexiao Tao, Jun Tu, Zhenshan Liu, Yufeng Yao, Wen Yang, Zhaoyuan Hou, Junling Liu, Xiao-Dong Yang, Qiang Zou, Bing Su, Zhiduo Liu, Jun Ni, Jinke Cheng, Xuefeng Wu

**Affiliations:** 1 Shanghai Institute of Immunology, Department of Immunology and Microbiology, Shanghai Jiao Tong University School of Medicine, Shanghai, 200025 China.; 2 Hongqiao International Institute of Medicine, Shanghai Tongren Hospital, Shanghai Jiao Tong University School of Medicine, Shanghai, 200025 China.; 3 State Key Laboratory of Oncogenes and Related Genes, Shanghai Jiao Tong University School of Medicine, Shanghai, 200025 China.; 4 Shanghai Key Laboratory for Tumor Microenvironment and Inflammation, Shanghai Jiao Tong University School of Medicine, Shanghai, 200025 China.; 5 Department of Biochemistry and Molecular Cell Biology, Shanghai Jiao Tong University School of Medicine, Shanghai, 200025 China.

**Keywords:** SIRT3, NLRC4, deacetylation, *S. typhimurium* infection, inflammasome

## Abstract

*Salmonella typhimurium* (*S. typhimurium*) infection of macrophage induces NLRC4 inflammasome-mediated production of the pro-inflammatory cytokines IL-1β. Post-translational modifications on NLRC4 are critical for its activation. Sirtuin3 (SIRT3) is the most thoroughly studied mitochondrial nicotinamide adenine dinucleotide (NAD^+^) -dependent deacetylase. We wondered whether SIRT3 mediated-deacetylation could take part in NLRC4 inflammasome activation.

**Methods:** We initially tested IL-1β production and pyroptosis after cytosolic transfection of flagellin or *S. typhimurium* infection in wild type and SIRT3-deficient primary peritoneal macrophages via immunoblotting and ELISA assay. These results were confirmed in SIRT3-deficient immortalized bone marrow derived macrophages (iBMDMs) which were generated by CRISPR-Cas9 technology. In addition,* in vivo* experiments were conducted to confirm the role of SIRT3 in *S. typhimurium*-induced cytokines production. Then NLRC4 assembly was analyzed by immune-fluorescence assay and ASC oligomerization assay. Immunoblotting, ELISA and flow cytometry were performed to clarify the role of SIRT3 in NLRP3 and AIM2 inflammasomes activation. To further investigate the mechanism of SIRT3 in NLRC4 activation, co-immunoprecipitation (Co-IP), we did immunoblot, cellular fractionation and in-vitro deacetylation assay. Finally, to clarify the acetylation sites of NLRC4, we performed liquid chromatography-mass spectrometry (LC-MS) and immunoblotting analysis.

**Results:** SIRT3 deficiency led to significantly impaired NLRC4 inflammasome activation and pyroptosis both *in vitro* and *in vivo*. Furthermore, SIRT3 promotes NLRC4 inflammasome assembly by inducing more ASC speck formation and ASC oligomerization. However, SIRT3 is dispensable for NLRP3 and AIM2 inflammasome activation. Moreover, SIRT3 interacts with and deacetylates NLRC4 to promote its activation. Finally, we proved that deacetylation of NLRC4 at Lys71 or Lys272 could promote its activation.

**Conclusions:** Our study reveals that SIRT3 mediated-deacetylation of NLRC4 is pivotal for NLRC4 activation and the acetylation switch of NLRC4 may aid the clearance of *S. typhimurium* infection.

## Introduction

The neuronal apoptosis inhibitory protein (NAIP)/NLR family caspase activation and recruitment domain-containing protein 4 (NLRC4) inflammasome is one of the best characterized inflammasomes during *S. typhimurium* infection 1-4. Activation of NLRC4 inflammasome by *S. typhimurium* depends largely on cytosolic expression of bacterial flagellin or type 3 secretion system (T3SS). Genetic, biochemical, and structural studies found that the NAIP subfamily of NLRs act as sensors to detect bacterial flagellin or T3SS proteins 5-9. NAIP5 and NAIP6 directly recognize flagellin, whereas NAIP2 recognizes T3SS rod proteins in mice. Activated NAIPs induce downstream oligomerization and activation of NLRC4, which act as an adaptor for the downstream recruitment and activation of caspase-1 10-12. Activation of caspase-1 initiates innate immune responses by cleaving of pro-IL-1β and pro-IL-18, and leading to their activation and release 1, 2. NAIP, NLRC4, and caspase-1 are sufficient to initiate pyroptosis, but processing IL-1β and IL-18 cytokines requires recruitment of the ASC adaptor to the complex 13, 14.

Post-translational modifications (PTMs) of proteins such as acetylation and phosphorylation are emerging as major regulatory mechanisms in all life forms 15, 16. Murine NLRC4 expressed in macrophages was found to be phosphorylated on Serine 533 (S533) in response to *Salmonella* exposure. And the δ isoform of Protein Kinase C (PKCδ) was found to phosphorylate NLRC4 *in vitro* 17. Recently, another kinase, Leucine Rich Repeat containing Kinase 2 (LRRK2), was also found to associate with NLRC4. Association of NLRC4 with LRRK2 was accompanied by phosphorylation on S533 18. Intriguingly, a recent paper reported that NLRC4 inflammasome activation is independent on phosphorylation during infection 19. Some of the differences in these results might derive from the use of varying C57CBL/6 mice 19. Nevertheless, roles of other PTMs, such as acetylation in regulating NLRC4 inflammasome still remains unknown and requires further investigation. Protein acetylation plays important roles in various biological processes, including immune responses 20. For example, SIRT2 mediated NLRP3 acetylation facilitates the activation of the NLRP3 inflammasome 21. Lysine acetylation occurs through lysine acetyltransferase (KAT)-catalyzed transfer of an acetyl group from acetyl-CoA to the ε-amino side chain of lysine. Acetylation is reversed by Zn^2+^-dependent histone deacetylases (HDACs), or by the NAD^+^-dependent sirtuin family of deacetylases, which means deacetylation 16, 22, 23. Sirtuins are a conserved family of proteins processing NAD^+^-dependent deacetylase activity 24. Among all the mitochondria sirtuins, SIRT3 is the most thoroughly studied one 25. It possesses robust deacetylase activity toward a cadre of metabolic targets, and taken part in mitochondria energy production, fuel partitioning, stress responses, and signaling 24, 26. Recent studies revealed that depletion of the mitochondrial-enriched sirtuin deacetylase SIRT3 increased NLRP3 inflammasome activation in association with excessive mitochondrial ROS production 27. By contrast, much less is understood about the functions of SIRT3 mediated deacetylation in NLRC4 inflammasome activation.

In the current study, we found that SIRT3 promotes NLRC4 inflammasome activation through deacetylating NLRC4. Acetylation at Lys71 or Lys272 of NLRC4 restraining its activation and *S. typhimurium* infection triggers SIRT3 to deacetylate and activate NLRC4.

## Materials and Methods

### Mice

SIRT3^-/-^ mice were obtained from Prof. Shimin Zhao's Laboratory (Fudan University) 28. All mice were bred and maintained in individually ventilated cages under specific pathogen-free conditions in accredited animal facilities. 8-10-wk-old gender matched littermate control mice were used as controls for all experiments. All animal procedures were performed in compliance with protocols approved by the Institutional Animal Care and Use Committee of Shanghai Jiao Tong University School of Medicine.

### Reagents and antibodies

LPS (Escherichia coli serotype 0111: B4), flagellin from* S. typhimurium*, and ATP (A2383) were purchased from Sigma-Aldrich. Nigericin was purchased from InvivoGen. Anti-caspase-1 p20, anti-ASC and NLRP3 were purchased from Adipogen. Anti-NLRC4 antibodies were from ECM Bioscience. Anti-mouse-IL-1β was purchased from GeneTex. Anti-SIRT3 and anti-pan-acetyl-lysine antibodies were from Cell Signaling Technology. Anti-flag and anti-HA antibodies were from Sigma-Aldrich. Anti-tubulin and anti-HSP90 antibodies were from Proteintech. Proteinase inhibitor was purchased from Roche. Deacetylase inhibitor from Beyotime. Recombinant SIRT3 peptide and DSS were from Sigma-Aldrich. Protein A/G magic beads from Sigma-Aldrich. DAPI, anti-mouse and anti-rabbit anti-Rat secondary Ab were from Thermo Fisher Scientific. All antibodies for Western Blot were used as a dilution of 1:1000. ATP 5a (14748) was from Abcam; Mitochondria isolation kit for cultured cells (89874) was from Thermo Scientific. MitoSOX, Mitotracker green, Mitotracker deep red were from Invitrogen. Antibody of GSDMD (ab 209845) was from Abcam. LDH cytotoxicity assay kit was from Promega.

### Cell culture

Unless otherwise indicated, all cells were cultured at 37 °C in 95% air and 5% CO_2_. Peritoneal macrophages were elicited by intraperitoneal injection of 1 mL BBL thioglycolate medium, brewer modified (4%; BD), and then they were recovered 4d later by peritoneal lavage with 20 mL PBS. The peritoneal macrophages were cultured in RPMI cell culture medium (Gibco) containing 10%FBS (Gibco), 1% penicillin and streptomycin. Primary BMDMs were grown for 6 days in DMEM supplemented with 10% FBS (Gibco), 30% medium conditioned by L929 mouse fibroblasts, and 1% penicillin and streptomycin. BMDMs in antibiotic-free medium were seeded onto 6-well plates at a density of 2×10^6 cells per well, followed by incubation overnight. L929 cells were cultured in RPMI-1640 medium supplemented with 10% FBS. 2 mM glutamine, 1% penicillin and streptomycin. HEK293T cells were cultured in DMEM supplemented with 10% FBS, 2mM glutamine and 1% penicillin and streptomycin.

### Salmonella typhimurium culture

*Salmonella enterica serovar typhimurium* SL1344, were inoculated into lysogeny broth (LB) and incubated overnight under aerobic conditions at 37 °C. *S. typhimurium* SL1344 were sub-cultured (1:100) for 3 h at 37 °C in fresh LB medium to induce SPI-1 expression.

### Plasmids

The plasmid SIRT3-HA expressing was a gift from W. Yang (Department of Biochemistry and Molecular Cell Biology, Shanghai Jiao Tong University School of Medicine, Shanghai, China). SIRT3 mutant H248Y construct was generated by site-directed point mutagenesis. Zizhen Kang (Shanghai Institute of Immunology and Department of Immunology and Microbiology, Shanghai Jiao Tong University School of Medicine, Shanghai, China) provided NLRC4 WT and pro-caspase-1 plasmids, which were re-cloned into pCDH-CMV-MCS-EF1-Puro vector.

### Stimulation of macrophages

To induce inflammasome activation, cells were plated in a 6-well plate overnight, and then the medium was changed to Opti-MEM the next morning before stimulation. For bacterial protein transfection, 0.5 mg, or 1 mg of ultrapure flagellin from *Salmonella typhimurium* were resuspended in Opti-MEM and mixed with 20 µL of DOTAP per reaction. The reaction mixture was incubated for 1 h and added to macrophages. For bacterial infection, *S. typhimurium* were used at an MOI of 20 for 2 h and 4 h of incubation. For activation of the canonical NLRP3 inflammasome, macrophages were primed for 4 h with 500 ng/mL ultrapure LPS followed by stimulating for 1 h with 5 mM ATP or nigericin (5 µM). For activation of AIM2 inflammasome, macrophages were transfected with 2 µg/mL poly (dA:dT). Cell culture supernatants were collected for enzyme-linked immunosorbent assays (ELISAs) or concentrated with methanol and chloroform for immunoblot.

### Cytokines measurements

Mice serum was collected and measured for IL-1β using ELISA kits according to de manufacturer's protocol. Supernatant from cell cultures were analyzed for mouse IL-1β and IL-6 with ELISA kits from BD. All procedures followed the manufacturer's instructions.

### Cytotoxicity assay

Cytotoxicity was measured via the activity of LDH released from macrophages as described 29. Macrophages were seeded in 96-well clear-bottom white-walled tissue culture-treated plates (Corning) at 10^5^ cells/well. Cells were then treated as before for NLRC4 inflammasome activation. Then the supernatants were collected and assayed for LDH activity according to the manufacturer's instructions.

### Animal infection

Frozen stocks of *S. typhimurium* were prepared from LB-grown *S. typhimurium*, quantified prior to infection, and diluted in PBS for infections. For acute *S. typhimurium* infection, mice were injected intraperitoneally with 10^7^ CFU in 200 µL PBS. After 6 h infection of *S. typhimurium*, serum and peritoneal macrophages were collected and measured for IL-1β using ELISA kit.

### Immunofluorescence staining

To detect the formation of ASC speck, mouse peritoneal macrophages were seeded on coverslips in 12-well plates. After *S. typhimurium* infection or transfection with flagellin, the cells were fixed with 4% paraformaldehyde for 15 min, permeabilized with 0.2% Triton X-100 for 10 min, and blocked in 3% BSA for 1 h. Cells were then incubated with primary antibodies (such as rabbit anti-ASC) overnight at 4 °C. Alex Fluor 488-conjugated secondary antibody was incubated for 1 h. Nuclei were stained with DAPI. Confocal micrographs were imaged using an Eclipse confocal microscope (Nikon).

### ASC oligomerization assay

For the generation of Triton X-100-soluble and insoluble fractions, differentially stimulated macrophages were lysed with TBS lysis buffer (50 mM Tris-HCL, PH 7.4, 150 mM NaCl containing 0.5% Triton X-100), EDTA free protease inhibitor cocktail, and phosphatase inhibitor cocktail. The lysates were centrifuged at 6800g for 15 min at 4 °C, and the pallet and supernatants were used as the Triton X-100 insoluble and -soluble fraction. For the detection of ASC oligomerization, the Triton X-100 insoluble pallets were washed with TritonX-100 TBS lysis buffer and then were resuspended in 200 µl PBS buffer (PBS PH 7.4, 150 mM NaCl without Triton X-100). The resuspended pallets underwent cross-linkage for 30 min at room temperature with 2 mM DSS (final concentration) then spun down at 6800g for 15 min at 4 °C. The pallets were dissolved with 35 µl 2X SDS sample buffer and subjected to immunoblotting.

### Immunoblotting and immunoprecipitation

For immunoblotting analysis, the total cell lysates were prepared in RIPA lysis buffer supplemented with complete protease inhibitors cocktail. Cell lysates were dissolved in SDS buffer and resolved by 7.5%-12% SDS-PAGE. After electrophoresis, separated proteins were transferred onto polyvinylidene fluoride (PVDF) membrane. The membrane was then blocked with 5% nonfat milk. After incubation with specific primary antibody, horseradish peroxidase-conjugated secondary antibody was applied. The positive immune reactive signals were visualized by enhanced chemiluminescence according to the manufacturer's instruction. To detect interaction between SIRT3 and NLRC4, cells were lysed in NP-40 lysis buffer (50 mM Tris PH7.6,150 mM NaCl, 1% vol/vol NP-40, and complete protease inhibitors), followed by centrifugation at 17000g for 10 min at 4 °C. The supernatants were immunoprecipitated with anti-SIRT3 or anti-Flag antibody for 12 h at 4 °C. The proteins bound by antibody were pulled down by protein A/G magnetic beads. The immunoprecipitants were washed three times with NP-40 buffer and boiled in 1× SDS-loading buffer for immunoblot analysis.

To detect NLRC4 acetylation, cells were lysed in NP-40 lysis buffer supplemented with complete protease inhibitors cocktail and pan-deacetylase inhibitors (TSA/NAM), followed by sonication and centrifugation at 17000g for 10 min at 4 °C. The supernatants were immunoprecipitated with NLRC4 antibody or Flag antibody for 12 h at 4 °C. The immunoprecipitants were washed three times with NP-40 buffer and boiled in 1× SDS-loading buffer for immunoblot analysis.

### Isolation of mitochondria

Macrophages mitochondria were isolated using commercially available mitochondria isolation kit (Thermo Scientific) according to the manufacturer's instructions.

### Flow cytometry analysis

Mitochondrial mass was measured by fluorescence levels upon staining with Mitotracker green and Mitotracker deep red at 50 nM for 30 min at 37 °C. Mitochondria-associated ROS levels were measured by staining cells with MitoSOX at 2.5 µM for 30 min at 37 °C. Cells were then washed with PBS solution and re-suspended in cold PBS solution containing 1% FBS for FACS analysis.

### Generation of SIRT3 knockout iBMDMs by CRISPR-Cas9-mediated genome editing

The immortalized macrophage line iBMDMs were provided by Prof. Zhengfan Jiang. Lentiviral CRISPR-Cas9 targeting guide RNA-expressing vector (lentiCRISPRv2) was obtained from Addgene. The SIRT3-knockout target sequence used was 5'-CACCGCTTTCAACAAACCTCCAGGG-3'. To generate SIRT3-knockout iBMDMs, lentiviruses containing the SIRT3 target sequence were used to transduce iBMDMs. Puromycin-positive iBMDMs were used by a limited dilution assay for single clones. Candidate knockout clones were screened by immunoblotting with anti-SIRT3 antibody.

### *In vitro* deacetylation

Deacetylation of NLRC4 by SIRT3 was performed by incubating purified recombinant SIRT3 and purified NLRC4 protein in the deacetylation buffer for 30 min at 30 °C. The deacetylation buffer including 50 mM Tris-HCL (PH 8.0), 130 mM NaCl, 2.5 mM KCl, 1 mM MgCl_2_ in the presence of 10mM NAM, and in the presence or absence of 1 mM NAD^+^. Purified recombinant SIRT3 protein was purchased from Invitrogen. Purified human SIRT3 (200 ng) was incubated with purified 3×Flag NLRC4 (50 ng) for deacetylation assay under the upper conditions. The reaction was stopped by the addition of SDS sample buffer and heating for 5 min at 95 °C. Protein were subjected to SDS-PAGE followed by immunoblotting for NLRC4 acetylation.

### NLRC4 protein purification

HEK293T cells transfected with plasmids expressing 3×Flag NLRC4 were lysed in NP-40 lysis buffer. The cell lysates were incubated with anti-Flag antibody and protein A/G magnetic beads at 4 °C. Beads were washed extensively with NP-40 lysis buffer with 0.5 mg/mL 3×Flag peptide in PBS.

### Identification of acetylation site by LC-MS/MS analysis

HEK293T cells were transfected with plasmids expressing Flag-tagged human NLRC4 with or without plasmid-encoding human SIRT3 using Lipofectamine 2000. Cell lysates were immunoprecipitated with anti-Flag and protein A/G magnetic beads. Proteins were resolved by SDS-PAGE, and bands corresponding with NLRC4 were excised, reduced, alkylated, and digested with *in situ* trypsin. Tryptic peptides were analyzed with a Q Exactive HF-mass spectrometer (ThermoFisher Scientifc).

### Quantification and statistical analysis

No statistical methods were used to estimate sample size. A standard two-tailed unpaired Student's t test was used for statistical analysis of two groups. Statistical analysis of survival curves was performed with a log-rank (Mantel-Cox) test. Statistical analyzed data are expressed as mean ± standard error of the mean (SEM). A p value < 0.05 is considered as statistically significant. We performed the statistical analyses using GraphPad Prism 7.0.

## Results

### SIRT3 deficiency impairs NLRC4 inflammasome activation

**Figure 1:**
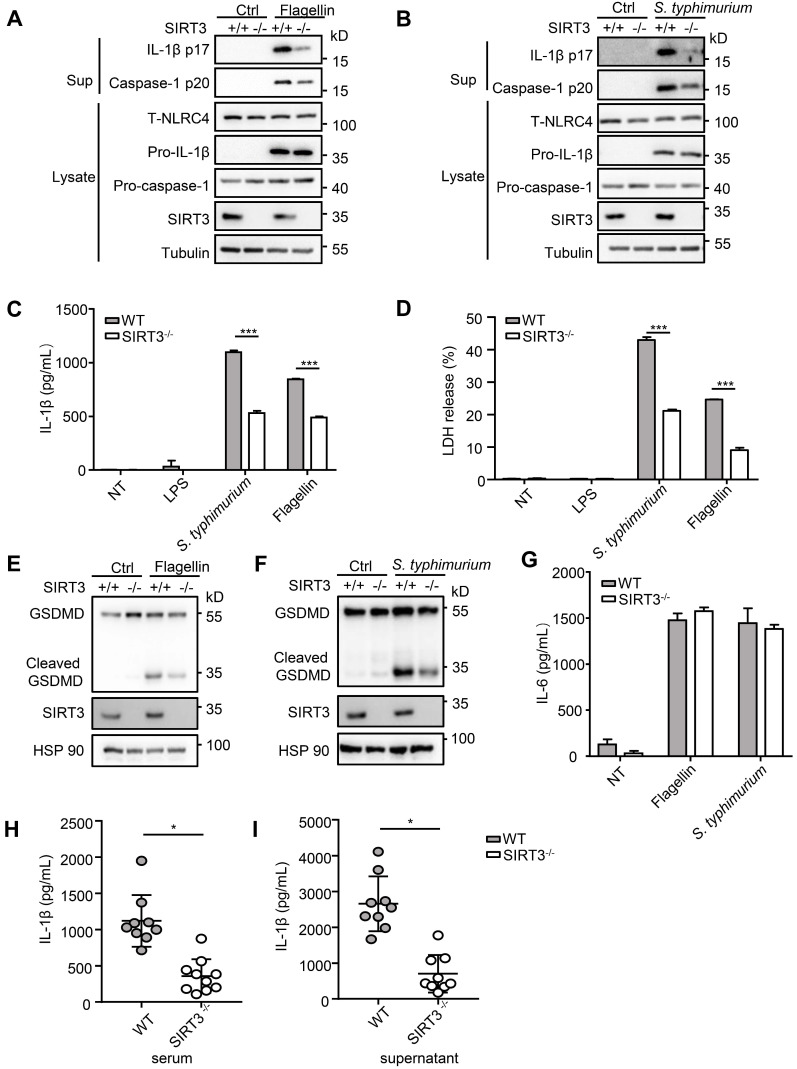
** SIRT3 deficiency impairs NLRC4 inflammasome activation.** (A) LPS-primed WT and SIRT3^-/-^ peritoneal macrophages were treated with 1 µg/mL flagellin for 6 h. Cell lysates and culture supernatants (Sup) were collected and immunoblotted with the indicated antibodies. (B) Peritoneal macrophages from littermate control (WT) and SIRT3^-/-^ mice were infected with *S. typhimurium* at an MOI of 20 for 2 h. Cell lysates and culture supernatants were collected and immunoblotted with the indicated antibodies. (C) ELISA of IL-1β in cell-free supernatants from WT and SIRT3^-/-^ peritoneal macrophages that were either pretreated with LPS (500 ng/mL) for 4 h followed by transfected with Flagellin (1 µg/mL) or infected with *S. typhimurium* at an MOI of 20 for 2 h. (D) LDH in cell free supernatants from WT and SIRT3^-/-^ peritoneal macrophages that were treated as stated in C. (E-F) Immunoblot analysis of full-length(53kD) and cleaved (30kD) GSDMD protein in lysate of macrophages treated as stated in C. (G) ELISA of IL-6 in cell free supernatants from WT and SIRT3^-/-^ peritoneal macrophages that were treated as stated in C. (H) ELISA analysis of IL-1β levels in sera of littermate control (WT n=9) and SIRT3^-/-^ mice (n=10) 6 h after *S. typhimurium* infection. (I) ELISA analysis of IL-1β from supernatants of overnight-cultured equal number of macrophages isolated from flushed peritoneal cells of WT and SIRT3^-/-^ mice which were infected by* S. typhimurium* for 6 h. Data are presented as means ± SEM; *, P < 0.05; ***, P <0.001 (Student's t test) all conditions were determined in technology triplicate. In each panel, data are representative of at least three independent experiments.

*S. typhimurium* infection of macrophages induces NLRC4 inflammasome-dependent cleavage of pro-caspase-1 (p45) into active caspase-1(p20 or p20/p10 heterodimer) and subsequently produced mature cytokines IL-1β and IL-18 8. To determine the role of SIRT3 in NLRC4 inflammasome activation, we first examined the cleavage of pro-caspase-1 and pro-IL-1β in response to defined NLRC4 inflammasome activators in WT and SIRT3-deficient macrophages (Figure 1A-C). Transfection of purified flagellin, a component of the intracellular pathogen* S. typhimurium*, induced robust pro-caspase-1 and pro-IL-1β cleavage in the WT but not the SIRT3-deficient macrophages (Figure 1A, C). Consistently, the SIRT3-deficient macrophages also showed reduced ability to activate pro-caspase-1 and pro-IL-1β as compared with the WT cells in response to *S. typhimurium* infection (Figure 1B, C). Besides cleaving pro-IL-Iβ, active caspase-1 also triggers a form of programmed necrosis known as pyroptosis. Pyroptosis features pore formation in the plasma membrane, cell swelling and rupture of the membrane, causing massive leakage of cytosolic contents 30. We found that in SIRT3-deficient macrophages *S. typhimurium* infection or flagellin cytosolic transfection resulted in a reduction in LDH release (Figure 1D), which is used as a measure of pyroptosis. During the pyroptosis, the active caspase-1 also cleaves the full-length gasdermin D to its active N-terminal fragment, this active gasdermin D acts as pyroptosis executioner and plays important role in mature IL-1β release. The reduction of caspase-1 in SIRT3-deficient macrophages blocked the cleavage of gasdermin D to its active N-terminal fragment in response to flagellin transfection or *S. typhimurium* infection (Figure 1E-F). Importantly, SIRT3-deficient macrophages resembled WT cells in the ability to produce IL-6 in response to NLRC4 inflammasome stimuli (Figure 1G), indicating that the defect is specific to NLRC4 inflammasome activation. We further investigated the contribution of SIRT3 to NLRC4 inflammasome activation by infecting mice *in vivo*. Recent studies reported that NLRC4-dependent IL-1β production plays important role in *S. typhimurium* infection 31-33. We injected WT and* SIRT3^-/-^* mice intraperitoneally with *S. typhimurium* and monitored the IL-1β levels in the serum of these mice 6 h after infection. Compared to WT mice, *SIRT3^-/-^* mice exhibited lower IL-1β production (Figure 1H). Moreover, the peritoneal macrophages sorted from infected *SIRT3^-/-^* mice produced smaller amount of IL-1β compared to WT controls (Figure 1I), suggesting impaired inflammasome activation in *SIRT3^-/-^* macrophages. Collectively, these data suggest that SIRT3 is specifically required for the optimal activation of the NLRC4 inflammasome* in vitro* and *in vivo*.

### SIRT3 promotes NLRC4 inflammasome activation in iBMDMs

**Figure 2:**
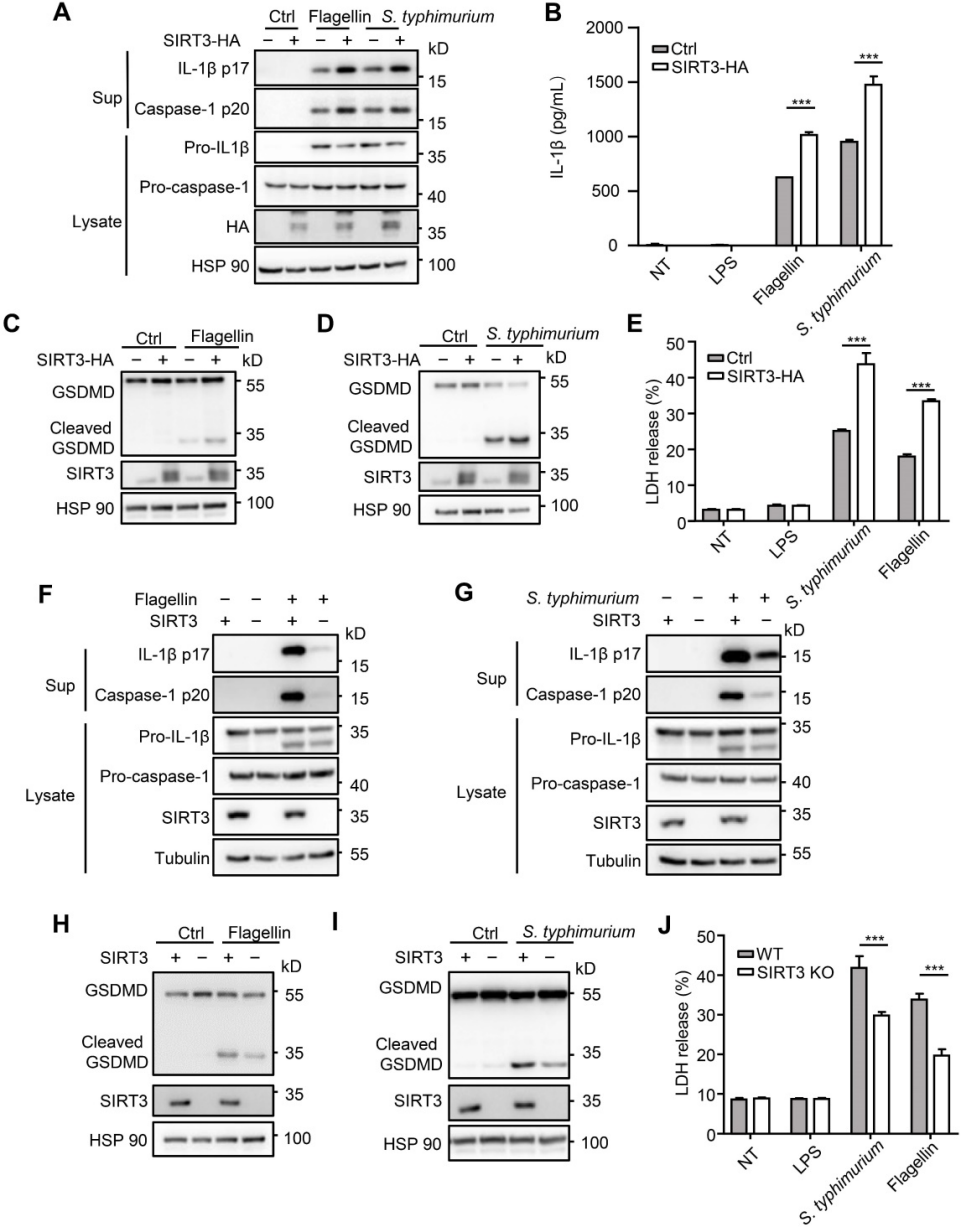
** SIRT3 promotes NLRC4 activation in iBMDMs.** (A) Immortalized bone marrow derived macrophages (iBMDMs) were transfected with control (Ctrl) or HA-tagged SIRT3. These cells were transfected with flagellin or infected with *S. typhimurium* at an MOI of 20 for 2 h, then the cell lysates and culture supernatants (Sup) were collected and immunoblotted with the indicated antibodies. (B) ELISA analysis of IL-1β from the cell-free supernatant of cells treated as stated in A. (C-D) Immunoblot analysis of full-length (53kD) and cleaved (30kD) GSDMD protein in lysate of iBMDMs that were treated as stated in A. (E) LDH release in cell free supernatants from iBMDMs that were treated as stated in A. (F-G) SIRT3-deficient iBMDMs were generated by CRISPR-Cas9-mediated deletion. These cells were primed with LPS followed by transfected with 1 μg/mL flagellin for 6 h (F) or infected with *S. typhimurium* at an MOI of 20 for 2 h (G). Cell lysate and culture supernatants were collected and immunoblotted with the indicated antibodies. (H-I) SIRT3-deficient iBMDMs were treated as stated in F and G. Cells were lysed and detected with antibody to GSDMD by immunoblot. (J) LDH in cell free supernatants from these SIRT3-deficient iBMDMs that were treated as stated in F-G. Data information: In B, E, J data are presented as means ± SEM; ***, P < 0.001 (Student's test). All the results are of at least three independent experiments. Each condition was performed in technology triplicate.

To further investigate role of SIRT3 in NLRC4 inflammasome action, we examined the consequence of overexpressing SIRT3. We found that HA-tagged SIRT3 expression in immortalized bone marrow derived macrophages (iBMDMs) led to increased caspase-1 cleavage, IL-1β production (Figure 2A-B), and promoted gasdermin D cleavage to its active N-terminal fragment (Figure 2C-D) and LDH release (Figure 2E). Consistent with primary macrophages, SIRT3 knockout iBMDMs generated by CRISPR-Cas9 also produced less IL-1β after flagellin transfection or *S. typhimurium* infection (Figure 2F-G). In addition, SIRT3 knockout iBMDMs showed lower gasdermin D cleavage (Figure 2H-I) and LDH release in response to NLRC4 inflammasome stimulators (Figure 2J). These data further prove that SIRT3 is pivotal for NLRC4 inflammasome activation.

### SIRT3 promotes ASC speck formation and assembly of NLRC4 inflammasome

**Figure 3:**
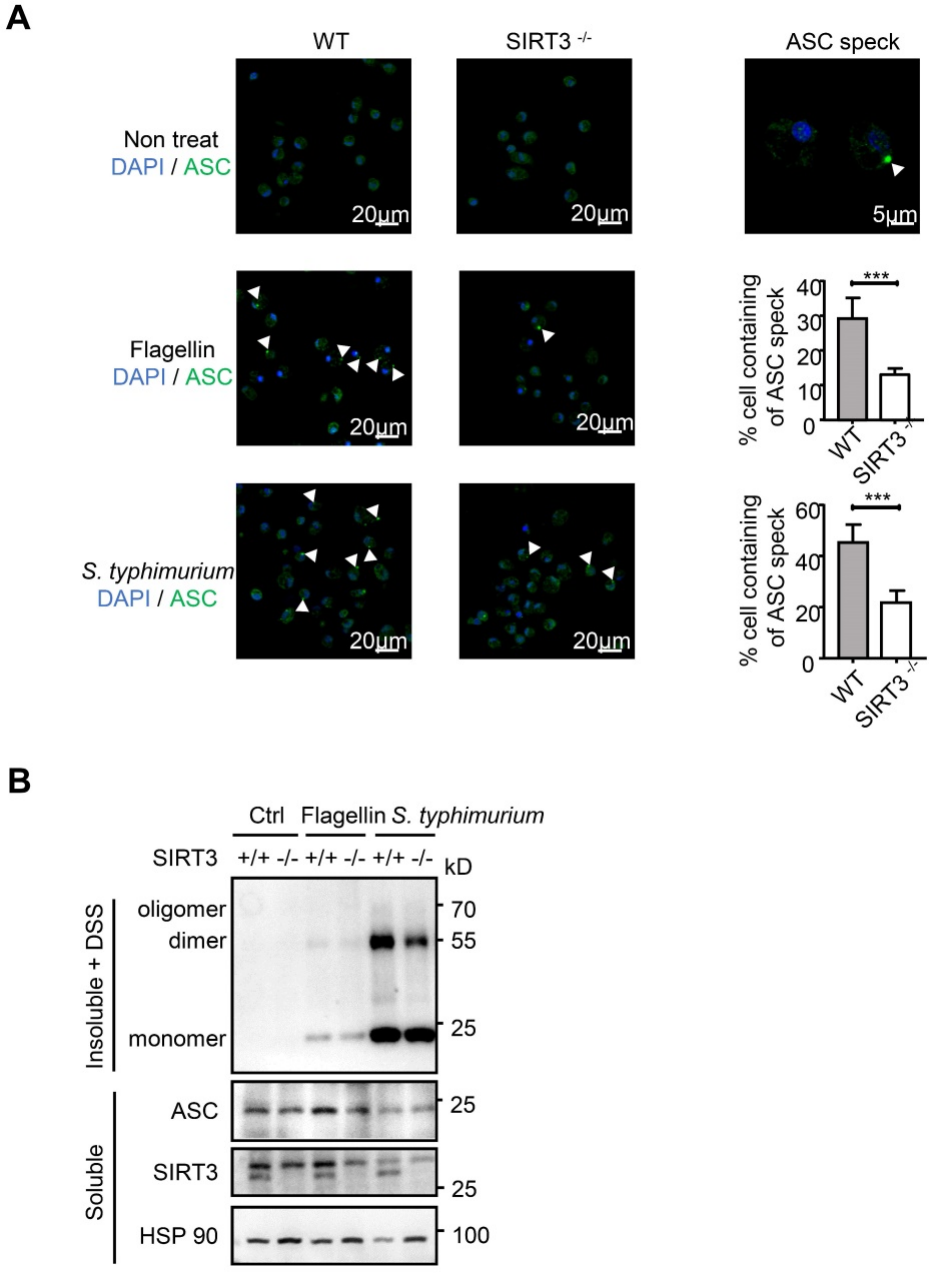
**SIRT3 promotes ASC speck formation and assembly of NLRC4 inflammasome.** (A) LPS-primed WT and SIRT3-/- peritoneal macrophages were either cytosolic transfected with 1 µg/mL flagellin or infected with *S. typhimurium* at an MOI of 20. ASC speck formation was assayed by ASC immunofluorescent staining, and cells were counterstained by DAPI (blue). Fluorescent images were analyzed by confocal microscopy. Percentages of macrophages containing ASC foci was quantified (right), with at least 200 cells counted in each experiment. Bars, 20 µm. Arrows in the figures indicate the ASC speck. (B) Peritoneal macrophages from littermate control (WT) and SIRT3-/- mice were cytosolic delivery of 1μg/mL flagellin or infected with *S. typhimurium* at an MOI of 20 for 2 h. Cells were dissolved with Triton X-100-containing buffer followed by cross-linkage of insoluble fractions with DSS to capture ASC oligomers. Immunoblots of those insoluble fractions (Insoluble + DSS) and soluble fractions were detected with an antibody to ASC. Data information: In A, data are shown as means ± SEM; ***, P < 0.001 (Student's test). All results are representative of three independent experiments.

The NLRC4 Inflammasome typically consists of three major components: the receptor/sensor, the adaptor ASC, and the effector caspase-1. Although NAIP, NLRC4, and caspase-1 are sufficient to initiate pyroptosis, processing of the IL-1β and IL-18 cytokines requires recruitment of the ASC adaptor into the complex 13, 14. *S. typhimurium* infection activates the inflammasome sensors, and triggers prion-like polymerization of ASC and its recruitment into large filamentous structures, which leads to the inflammatory activity caspase-1 34, 35. In resting cells, ASC displays soluble cytoplasmic and nuclear localization, but, upon assembly of inflammasomes, it is mobilized to form a large singular paranuclear ASC “speck” (~1 μm in diameter) 36-38. To address how SIRT3 regulates NLRC4 inflammasome, we visualized the endogenous ASC specks in macrophages treated with purified flagellin or infected with *S. typhimurium* using immunofluorescence staining (Figure 3A). Under non-treated condition, wild-type and SIRT3-deficient macrophages ASC showed soluble cytoplasmic and nuclear localization. Upon *S. typhimurium* treatment, ASC in activated cells with inflammasome assembly mobilized to form a large singular ASC speck (Figure 3A right panel). However, the frequency of ASC speck-containing cells decreased by 60% in SIRT3-deficient macrophages compared with that in WT controls (Figure 3A right panel), suggesting SIRT3 is required for ASC speck formation upon NLRC4 inflammasome activation.

Biochemical studies have demonstrated that ASC specks are oligomers of ASC protein that manifest as Triton X-100-insoluble aggregates 18, 38, 39. We prepared Triton X-100-soluble and Triton X-100-insoluble fractions from WT and SIRT3-deficient macrophages transfected with flagellin or infected with *S. typhimurium* (Figure 3B). The Triton X-100-insoluble fractions were treated with the cross-linking agent disuccinimidyl substrate (DSS) to analyze the oligomerization of ASC. Consistent with decreased ASC speck formation (Figure 3A) in SIRT3-deficient macrophages, the formation of ASC dimer and oligomer in SIRT3-deficient macrophages was dramatically reduced compared with that in WT controls (Figure 3B). Collectively, these data indicate that SIRT3 promotes the formation of ASC specks in response to NLRC4 inflammasome activation.

### SIRT3 is dispensable for NLRP3 and AIM2 inflammasome activation

**Figure 4:**
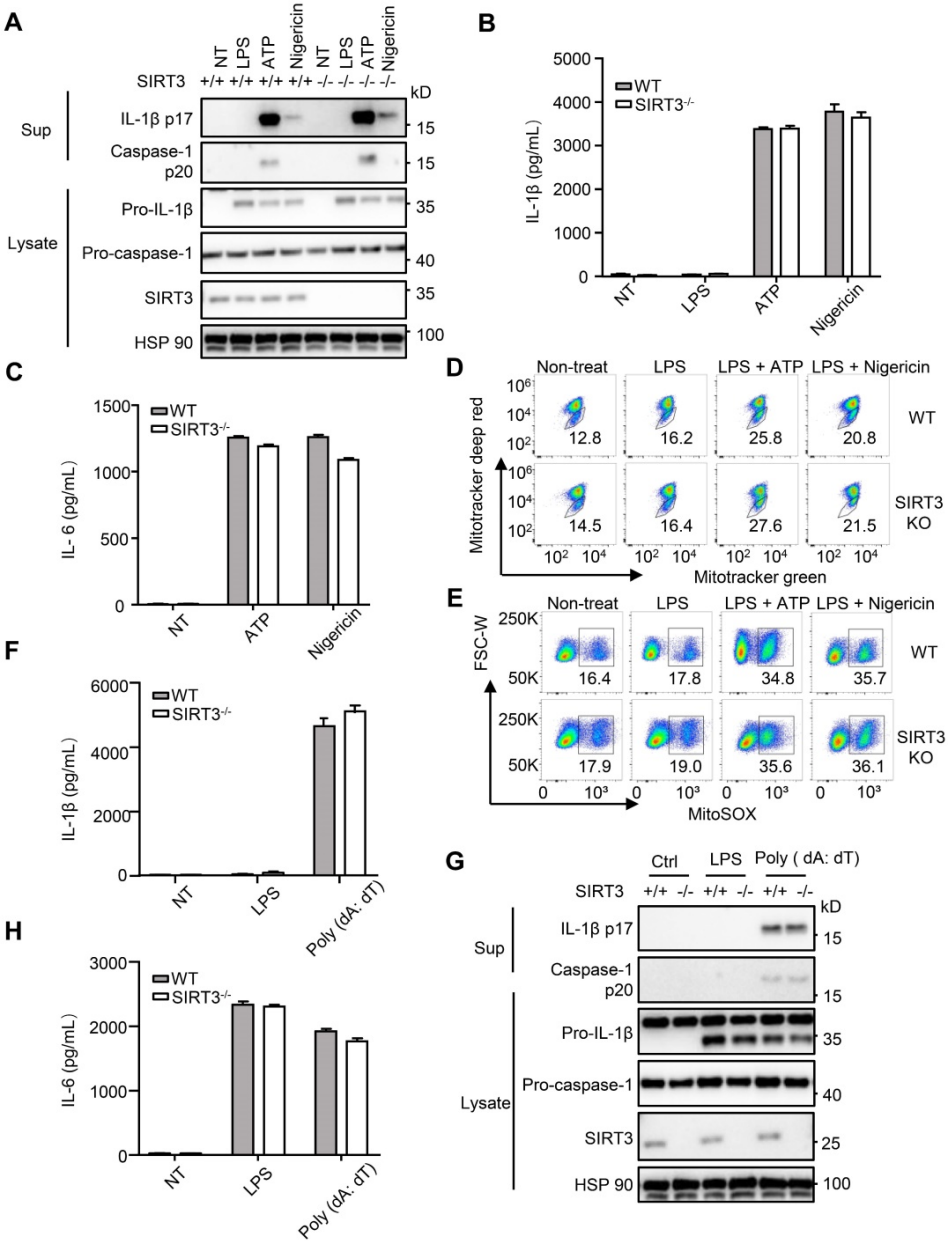
** SIRT3 is dispensable for NLRP3 and AIM2 inflammasome activation.** (A) WT and SIRT3-/- peritoneal macrophages were primed with 500 ng/mL LPS for 4 h and then treated with ATP (5 mM) or nigericin (5 μM) for 1 h. Cell lysates and culture supernatants were collected and immunoblotted with the indicated antibodies. (B-C) ELISA of IL-1β (B) and IL-6 (C) in cell-free supernatants from WT and SIRT3-/- peritoneal macrophages that were treated as (A). (D-E) Peritoneal macrophages were primed with LPS for 4 h followed by activated by ATP (5 mM) or nigericin (5 μM) for 1 h and then stained with Mitotracker green and Mitotracker deep red (D) or MitoSOX (E) for 30 min and analyzed by flow cytometry. (F) WT and SIRT3-/- peritoneal macrophages were primed with 500 ng/mL LPS for 4 h and then transfected with poly (dA: dT) (2 μg/mL) for 6 h. ELISA analysis of IL-1β in cell-free supernatants from these cells. (G) Cells were treated as (F), cell lysates and culture supernatants were collected and immunoblotted with the indicated antibodies. (H) ELISA of IL-6 in cell-free supernatants from WT and SIRT3-/- peritoneal macrophages that were treated as (F). Data information: In B-C, F and H, data are presented as means ± SEM. Data are representative of three independent experiments.

Upon *S. typhimurium* infection, both NLRC4 and the NACHT, LRR and PYD domains-containing protein 3 (NLRP3) inflammasomes are activated 14, 40. During *S. typhimurium* infection, these two NLRs resulted in the formation of a single ASC speck per macrophage. The activated NLRC4 recruits NLRP3 and NLRP3 interacts with the NACHT domain in NLRC4 40. NLRC4 located in a ring-like structure, whereas NLRP3 formed a smaller and more centralized ring-like structure inside NLRC4 14 . We next examined whether SIRT3 is involved in the activation of NLRP3 inflammasome. The NLRP3 inflammasome is a unique innate immune sensor that can be activated by a diverse array of endogenous metabolic signals to induce inflammation 41, 42. Activation of the NLRP3 inflammasome requires two signals. The first signal, i.e., the transcriptional priming, represents a critical checkpoint: once primed, the NLRP3 inflammasome is activated by an astonishing array stimuli, such as ATP and nigericin 43-45. We did not detect noticeable differences between WT and SIRT3-deficient macrophages in their ability to activate NLRP3 inflammasome (Figure 4A-B and Figure S1A). SIRT3-deficient and WT macrophages showed similar ability to produce IL-6 in response to NLRP3 inflammasome stimulation (Figure 4C). A wide variety of danger signals activate the NLRP3 inflammasome. One of the models proposes that NLRP3 is influenced by ROS 46, 47, for which mitochondria being the main source. SIRT3 had been shown to suppress mitochondrial ROS production under some conditions 33, 48, 49. To investigate a possible implication of SIRT3-mediated mitochondria condition in NLRP3 inflammasome activation, we measured ROS production during NLRP3 inflammasome activation in SIRT3-deficient macrophages. In agreement with comparable NLRP3 inflammasome activation, the SIRT3-deficient macrophages showed similar ROS production compared with WT macrophages after ATP or nigericin treatment (Figure 4D-E). This was determined using three types of mitochondria-specific labels that distinguish respiring (Mitotracker deep red), total (Mitotracker green) and ROS-generating mitochondria (MitoSOX) (Figure 4D-E). Collectively, these results suggest that SIRT3-deficient and WT macrophages had comparable mitochondrial ROS production during NLRP3 inflammasome activation and SIRT3 is dispensable for NLRP3 inflammasome activation.

AIM2 is another kind of inflammasome that detects the double-stranded DNA in cytosol 50, 51. To investigate the requirement of SIRT3 in response to poly (dA: dT) mediated AIM2 inflammasome activation, we transfected poly (dA: dT) into WT and SIRT3-deficient macrophages. IL-1β levels, caspase-1 activation (Figure 4F-G and Figure S1B), and IL-6 production (Figure 4H) in poly (dA: dT) transfected macrophages from WT or SIRT3-deficient mice were comparable. Collectively, these data suggest that SIRT3 is dispensable for AIM2 inflammasome activation.

### SIRT3 deacetylates NLRC4 to promote NLRC4 inflammasome activation

**Figure 5:**
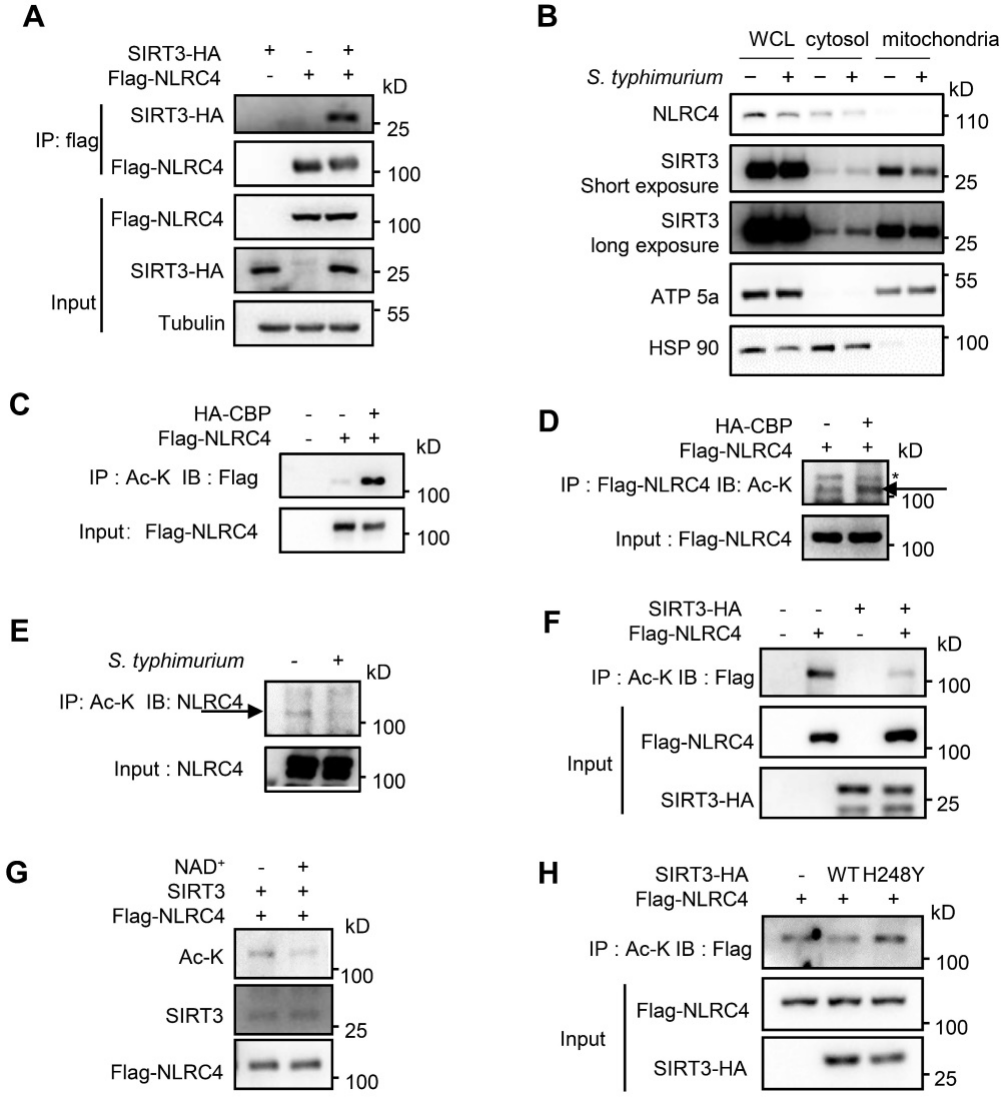
** SIRT3 interacts with and deacetylates NLRC4 to promote its activation.** (A) HEK293T cells were transfected with HA-tagged SIRT3 along with or without Flag-tagged NLRC4. Cell lysates were immunoprecipitated with anti-Flag and analyzed by immunoblot. (B) Subcellular distribution of NLRC4 and SIRT3 before and after *S. typhimurium* infection in peritoneal macrophages. ATP5a as a mitochondria marker. HSP 90 serves as cytosol marker. (C and D) HEK293T cells ectopically expressing NLRC4 were co-transfected with or without CBP. Cell lysates were then immunoprecipitated (IP) using pan-acetyl-lysine antibody (C) or anti-Flag NLRC4 antibody (D) and were immunoblotted with the indicated antibodies. Arrows in D indicate the Ac-NLRC4 band, * in D indicates non-specific band. (E) Peritoneal macrophages were infected with *S. typhimurium* at an MOI of 20 for 2 h. Uninfected cells were included as controls. Cell lysates were then immunoprecipitated (IP) using pan-acetyl-lysine antibody and were immunoblotted with the indicated antibodies. Arrow indicates the NLRC4 band. (F) HEK293T cells were transfected with Flag-tagged NLRC4 along with or without HA-tagged SIRT3. Whole-cell lysates were then immunoprecipitated (IP) using pan-acetyl-lysine and were immunoblotted with the indicated antibody. (G) SIRT3 deacetylates NLRC4* in vitro*. NLRC4 (0.2 mg/μL) was purified and incubated with SIRT3 (0.1 mg/mL) with or without NAD^ +^ (1 mM). The acetylation levels were determined by Western blotting. (H) HEK293T cells were transfected with WT or H248Y mutant SIRT3 along with NLRC4. Cell lysates were then immunoprecipitated (IP) using pan-acetyl-lysine and were immunoblotted with the indicated antibodies. Data information: All the results are representative of at least three independent experiments.

The reduced ASC speck formation in SIRT3-deficient macrophages (Figure 3A) indicated that ASC oligomerization was impaired in the absence of SIRT3 during NLRC4 inflammasome activation. Biochemical evidence showed that formation of ASC oligomers was triggered and subsequently activated NLRC4 protein. Thus, interaction between SIRT3 and NLRC4 in the inflammasome pathways was analyzed. Co-immunoprecipitation experiments indicated that SIRT3 formed a complex with NLRC4 (Figure 5A). Cell fractionation confirmed that SIRT3 mainly located in mitochondria under resting conditions, while upon *S. typhimurium* infection, part of SIRT3 relocates into the cytosol, where it can interact and deacetylate NLRC4 (Figure 5B). As SIRT3 is a NAD^+^-dependent deacetylase, we hypothesize that SIRT3 exerts its deacetylase function through deacetylating NLRC4. We first tested whether NLRC4 can be acetylated. In order to maximize acetylation of NLRC4, we co-transfected NLRC4 with one of the Histone Acetyltransferase (HAT) CBP 52. As expected, CBP obviously promoted NLRC4 acetylation (Figure 5C-D). To examine the role of NLRC4 acetylation in NLRC4 inflammasome activation, we treated peritoneal macrophages with* S. typhimurium* and observed that acetylation of NLRC4 was reduced after *S. typhimurium* treatment (Figure 5E), suggesting that *S. typhimurium* leads to NLRC4 deacetylation. Co-transfected NLRC4 with WT SIRT3 in HEK 293T cells also showed decreased acetylation of NLRC4 (Figure 5F). To confirm whether NLRC4 is a substrate of NAD^+^-dependent deacetylase SIRT3, we purified NLRC4 and incubated it with recombinant SIRT3 at the presence of NAD^+^. The result showed that purified NLRC4 was indeed deacetylated by SIRT3 (Figure 5G). The enzymatic activity of SIRT3 is critical for the deacetylation of NLRC4 as SIRT3 H248Y mutant (activity null mutant) 53 was unable to induce NLRC4 deacetylation (Figure 5H). Collectively, these data suggest that acetylation inhibits NLRC4 activity and that SIRT3-mediated deacetylation of NLRC4 promotes NLRC4 activation upon* S. typhimurium* infection.

### NLRC4 K71 and K272 acetylation inhibits NLRC4 activation

**Figure 6:**
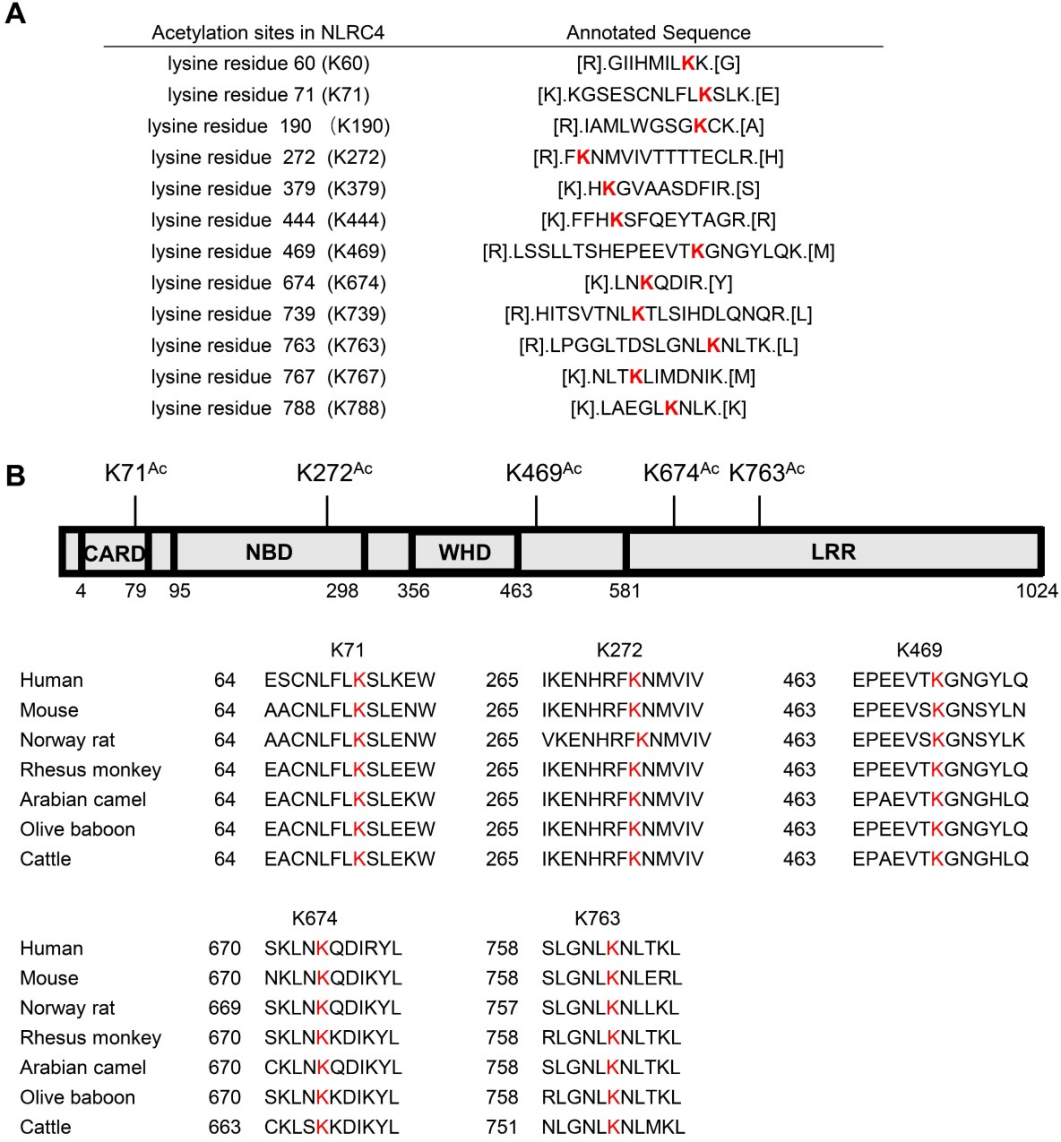
**Identification of NLRC4 acetylation.** (A) List of the identified acetylation Sites of NLRC4. HEK293T cells were transfected with Flag-tagged NLRC4, these cells were harvested and NLRC4 was immunoprecipitated and analyzed by mass spectrometer. (B) NLRC4 acetylation conserved sites. The sequences of NLRC4 in seven species were aligned. K71, K272, K469, K674, K763 of NLRC4 were highlighted in red.

**Figure 7:**
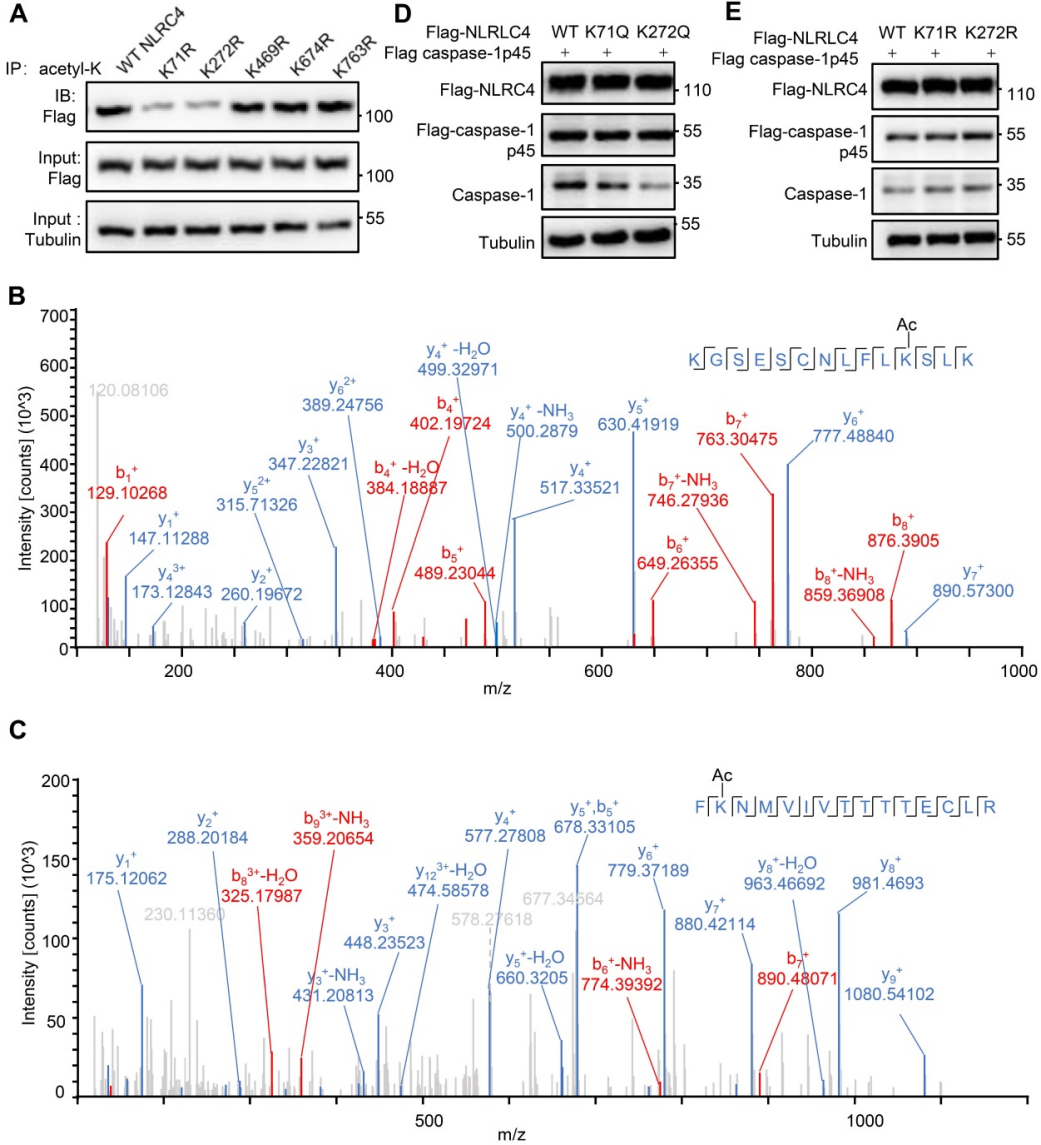
** Deacetylation at K71/K272 enhances NLRC4 activation.** (A) Flag-tagged NLRC4 and its mutants were transiently expressed in HEK293T cells, and lysates were subjected to immunoprecipitated using pan-acetyl-lysine antibody followed by Western blotting. (B-C) The MS/MS spectrum of modified peptides that include K71 and K272. (D-E) HEK293T cells were transfected with Flag-tagged WT NLRC4 and its acetylation-mimetic (K-to-Q) mutants (D) or K-to-R mutants (E) along with Flag-tagged-pro-caspase-1. Whole-cell lysates were analyzed by Western blotting for the presence of cleaved caspase-1. All results are representative of three independent experiments.

As SIRT3 mediated deacetylation of NLRC4 is involved in the regulation of NLRC4 activity (Figure 5), next we set out to investigate the acetylation of NLRC4 sites in quiescent cells. To do so, we first created a flag-NLRC4 stable HEK293T cell line and examined NLRC4 acetylation by performing liquid chromatography-mass spectrometry (LC-MS) with the immunoprecipitated Flag-NLRC4. We identified twelve lysine (K) acetylation sites of NLRC4: K60, K71, K190, K272, K379, K444, K469, K674, K739, K763, K767, and K788 (Figure 6A). Interestingly, five of these twelve sites are conserved across species (Figure 6B). To study the function of these sites, we mutated these conserved sites by replacing a lysine with arginine (R), which mimics the deacetylation state on lysine 54, and tested the effect of these mutations on NLRC4 acetylation in HEK293T cells. Mutating the K71 and K272 to R decreased the acetylation level of NLRC4 (Figure 7A). These data primarily showed that lysine (K) residue 71 and 272 were the major acetylation site of NLRC4 as LC-MS/MS analysis (Figure 7B-C). To confirm the function of acetylation of these two sites, we tested the effect of these mutations on NLRC4 activity in HEK293T cells that transfected along with pro-caspase-1. The NLRC4 activity can be reflected by the cleavage of the pro-caspase-1 55. We found that K-to-Q mutation of these two sites K71, K272 lead to a reduction in NLRC4 activity (Figure 7D). We further found that K-to-R mutation of K71 or K272 did not affect the cleavage of pro-caspse-1(Figure 7E). Taken together, these data suggest that acetylation of NLRC4 at K71 or K272 may inhibit NLRC4 activation.

## Discussion

As part of the innate immune response, NLRC4 inflammasome is activated by a range of intracellular bacteria, such as *S. typhimurium* 56, 57. However, regulation of NLRC4 in response to *S. typhimurium* still remains elusive 17, 18. In this study, we demonstrate that SIRT3-mediated deacetylation of NLRC4 promotes its activation in response to* S. typhimurium* infection.

Sirtuin family members modify multiple proteins therefore exert a concerted cellular functions 58. SIRT1 was reported to inhibit NLRP3 inflammasome 59, 60. Furthermore, the deacetylation ability of SIRT1 on the transcription factors, such as nuclear factor kappa B (NF-κB) may attribute to its suppressive role on inflammation 61, 62. Recently, a new finding indicates that SIRT2 deacetylates NLRP3 to suppress NLRP3 inflammasome assembly 21. Another report showed that SIRT2 also regulates tubulin rearrangement to inhibit NLRP3 inflammasome action 63. As the most studied mitochondria sirtuin member, SIRT3 is considered a ROS suppressor mainly by the deacetylation of the major mitochondrial antioxidant enzymes, such as isocitrate dehydrogenase 2, superoxide dismutase, and glutathione peroxidase 64, 65. The decreased production of ROS has been shown to inhibit NLRP3 inflammasome 33, 48. However, the role of SIRT3 in NLRC4 inflammasome had not been studied. Activation of NLRC4 inflammasome in mouse macrophages requires, in part, phosphorylation of S533 by kinase PKC 17, or leucine-rich repeat kinase (LRRK2) 18. How acetylation, the important post-translational modification regulates NLRC4 inflammasome activation is little known. Here, we revealed the molecular link between SRT3-mediated deacetylation and NLRC4 activation. We found that SIRT3 promotes *S. typhimurium* induced NLRC4 inflammasome activation. Importantly, SIRT3 specifically regulates NLRC4, but not NLRP3 or AIM2 inflammasomes. Our finding that SIRT3 is dispensable for NLRP3 inflammasome activation is somehow different from others 33, 48. This discrepancy can be due to different experimental conditions. We used a two-step protocol as described 66, 67, in contrast, a one-step protocol, i.e. LPS only was used in the studies which show SIRT3 inhibits NLRP3 inflammasome activation 68. Furthermore, in the same study cells were treated with 3 ng/mL LPS was for 24 h, in our experiments, we primed macrophages with 500 ng/mL LPS for 4 h. These different stimulations may the key course of different conclusions.

We identified lysine 71 or 272 of NLRC4 as the major acetylation sites, whose mutation to acetylation mimetic reduced NLRC4-mediated pro-caspase-1 cleavage (Figure 7D). However, further studies are required to establish: (1) whether modification at these sites is functionally relevant with regard to inflammasome activation by structure analysis; (2) whether there are other targets for deacetylation along this pathway; (3) cross-talks between acetylation and phosphorylation or other types of PTMs; (4) involvement of other deacetylases in NLRC4 deacetylation and activation, as previous studies showed that SIRT1 and SIRT2 took part in NLRP3 inflammasome activation, and NLRC4 recruits NLRP3 upon *S. typhimurium* infection. These two sirtuins may also take part in *S. typhimurium*-induced NLRC4 inflammasome.

Till now, *Salmonella enterica* is still a leading cause of community-acquired bloodstream infections in many low- and middle-income countries 69, 70. Our data showed that the effect of SIRT3 on NLRC4 activation might contribute to the clearance of *S. typhimurium* infection. We propose that SIRT3-mediated NLRC4 deacetylation increasing the assembly of NLRC4 inflammasome, thereby promoting the activation of NLRC4 inflammasome and production of IL-1β. In conclusion, the acetylation switch of NLRC4 may aid the clearance of *S. typhimurium* infection and SIRT3 might be a potential therapeutic target to fight against *S. typhimurium* infection.

## Supplementary Material

Supplementary figure S1.

## Abbreviations

AIM2: absent in melanoma 2
ASC: apoptosis-associated speck-like protein containing a CARD
ATP: adenosine triphosphate
BMDMs: bone marrow derived macrophages
Caspase-1: cysteinyl aspartate specific proteinase-1
CFU: colony-forming units
DAPI: 4,6-diamidino-2-phenylindole
DSS: disuccinimidyl suberate
EDTA: ethylene diamine tetra acetic acid
FBS: fetal bovine serum
HDAC: histone deacetylases
iBMDMs: immortalized bone marrow derived macrophage
IL-1β/6/18: interleukin-1β/6/18
KAT: lysine acetyltransferase
LB: lysogeny broth
LPS: lipopolysaccharide
LRRK2: leucine rich repeat containing kinase 2
MOI: multiplicity of infection
NAD+: nicotinamide adenine dinucleotide
NAIP2/5/6: neuronal apoptosis inhibitory protein 2/5/6
NLR: NOD-like receptor
NLRC4: NLR family activation and recruitment domain-containing protein 4
NLRP3: NOD-like receptor, pyrin domain containing protein 3
PBS: phosphate buffer saline
PKC δ: δ isoform of protein kinase C
PTM: post-translational modification
PVDF membrane: polyvinylidene fluoride (PVDF) membrane
ROS: reactive oxygen species
RPMI-1640: Roswell Park Memorial Institute-1640 medium
SDS-PAGE: sodium dodecyl sulfate- polyacrylamide gel electrophoresis
SIRT2/3: Sirtuin 2/3
SPI-1: Salmonella pathogenicity island 1
T3SS: Type III secretion system

## References

[1] Lamkanfi M, Dixit VM (2014). Mechanisms and functions of inflammasomes. Cell.

[2] Rathinam VA, Fitzgerald KA (2016). Inflammasome complexes: emerging mechanisms and effector functions. Cell.

[3] Poyet JL, Srinivasula SM, Tnani M, Razmara M, Fernandes-Alnemri T, Alnemri ES (2001). Identification of Ipaf, a human caspase-1-activating protein related to Apaf-1. J Biol Chem.

[4] Ren J, Sang Y, Tan Y, Tao J, Ni J, Liu S (2016). Acetylation of lysine 201 inhibits the DNA-binding ability of PhoP to regulate Salmonella virulence. PLoS Pathog.

[5] Franchi L, Amer A, Body-Malapel M, Kanneganti TD, Ozoren N, Jagirdar R (2006). Cytosolic flagellin requires Ipaf for activation of caspase-1 and interleukin 1beta in salmonella-infected macrophages. Nat Immunol.

[6] Miao EA, Mao DP, Yudkovsky N, Bonneau R, Lorang CG, Warren SE (2010). Innate immune detection of the type III secretion apparatus through the NLRC4 inflammasome. Proc Natl Acad Sci U S A.

[7] Miao EA, Alpuche-Aranda CM, Dors M, Clark AE, Bader MW, Miller SI (2006). Cytoplasmic flagellin activates caspase-1 and secretion of interleukin 1beta via Ipaf. Nat Immunol.

[8] Mariathasan S, Newton K, Monack DM, Vucic D, French DM, Lee WP (2004). Differential activation of the inflammasome by caspase 1 adaptors ASC and Ipaf. Nature.

[9] Zamboni DS, Kobayashi KS, Kohlsdorf T, Ogura Y, Long EM, Vance RE (2006). The Birc1e cytosolic pattern-recognition receptor contributes to the detection and control of Legionella pneumophila infection. Nat Immunol.

[10] Hu Z, Zhou Q, Zhang C, Fan S, Cheng W, Zhao Y (2015). Structural and biochemical basis for induced self-propagation of NLRC4. Science.

[11] Zhang L, Chen S, Ruan J, Wu J, Tong AB, Yin Q (2015). Cryo-EM structure of the activated NAIP2-NLRC4 inflammasome reveals nucleated polymerization. Science.

[12] Tenthorey JL, Haloupek N, Lopez-Blanco JR, Grob P, Adamson E, Hartenian E (2017). The structural basis of flagellin detection by NAIP5: A strategy to limit pathogen immune evasion. Science.

[13] Man SM, Tourlomousis P, Hopkins L, Monie TP, Fitzgerald KA, Bryant CE (2013). Salmonella infection induces recruitment of Caspase-8 to the inflammasome to modulate IL-1beta production. J Immunol.

[14] Man SM, Hopkins LJ, Nugent E, Cox S, Gluck IM, Tourlomousis P (2014). Inflammasome activation causes dual recruitment of NLRC4 and NLRP3 to the same macromolecular complex. Proc Natl Acad Sci U S A.

[15] Cohen P (2000). The regulation of protein function by multisite phosphorylation - a 25 year updat. Trends Biochem Sci.

[16] Verdin E, Ott M (2015). 50 years of protein acetylation: from gene regulation to epigenetics, metabolism and beyond. Nat Rev Mol Cell Biol.

[17] Qu Y, Misaghi S, Izrael-Tomasevic A, Newton K, Gilmour LL, Lamkanfi M (2012). Phosphorylation of NLRC4 is critical for inflammasome activation. Nature.

[18] Liu W, Liu X, Li Y, Zhao J, Liu Z, Hu Z (2017). LRRK2 promotes the activation of NLRC4 inflammasome during Salmonella Typhimurium infection. J Exp Med.

[19] Tenthorey JL, Chavez RA, Thompson TW, Deets KA, Vance RE, Rauch I (2020). NLRC4 inflammasome activation is NLRP3- and phosphorylation-independent during infection and does not protect from melanoma. J Exp Med.

[20] Shakespear MR, Halili MA, Irvine KM, Fairlie DP, Sweet MJ (2011). Histone deacetylases as regulators of inflammation and immunity. Trends Immunol.

[21] He M, Chiang HH, Luo HZ, Zheng ZF, Qiao Q, Wang L (2020). An acetylation switch of the NLRP3 inflammasome regulates aging-associated chronic inflammation and insulin resistance. Cell Metab.

[22] Narita T, Weinert BT, Choudhary C (2019). Functions and mechanisms of non-histone protein acetylation. Nat Rev Mol Cell Biol.

[23] Sabari BR, Zhang D, Allis CD, Zhao Y (2017). Metabolic regulation of gene expression through histone acylations. Nat Rev Mol Cell Biol.

[24] Verdin E, Hirschey MD, Finley LW, Haigis MC (2010). Sirtuin regulation of mitochondria: energy production, apoptosis, and signaling. Trends Biochem Sci.

[25] Zhang J, Xiang HG, Liu J, Chen Y, He RR, Liu B (2020). Mitochondrial Sirtuin 3: New emerging biological function and therapeutic target. Theranostics.

[26] Kumar S, Lombard DB (2015). Mitochondrial sirtuins and their relationships with metabolic disease and cancer. Antioxid Redox Signal.

[27] Traba J, Kwarteng-Siaw M, Okoli TC, Li J, Huffstutler RD, Bray A (2015). Fasting and refeeding differentially regulate NLRP3 inflammasome activation in human subjects. J Clin Invest.

[28] Hallows WC, Yu W, Smith BC, Devries MK, Ellinger JJ, Someya S (2011). Sirt3 promotes the urea cycle and fatty acid oxidation during dietary restriction. Mol Cell.

[29] Lightfield KL, Persson J, Brubaker SW, Witte CE, von Moltke J, Dunipace EA (2008). Critical function for Naip5 in inflammasome activation by a conserved carboxy-terminal domain of flagellin. Nat Immunol.

[30] Shi JJ, Zhao Y, Wang K, Shi XY, Wang Y, Huang HW (2015). Cleavage of GSDMD by inflammatory caspases determines pyroptotic cell death. Nature.

[31] Rao S, Schieber AMP, O'Connor CP, Leblanc M, Michel D, Ayres JS (2017). Pathogen-mediated inhibition of anorexia promotes host survival and transmission. Cell.

[32] Karki R, Lee E, Place D, Samir P, Mavuluri J, Sharma BR (2018). IRF8 regulates transcription of Naips for NLRC4 inflammasome activation. Cell.

[33] Liu PH, Huang GJ, Wei T, Gao J, Huang CL, Sun MW (2018). Sirtuin 3-induced macrophage autophagy in regulating NLRP3 inflammasome activation. Biochim Biophys Acta Mol Basis Dis.

[34] Cai X, Chen JQ, Xu H, Liu SQ, Jiang QX, Halfmann R (2014). Prion-like polymerization underlies signal transduction in antiviral immune defense and inflammasome activation. Cell.

[35] Lu A, Magupalli VG, Ruan J, Yin Q, Atianand MK, Vos MR (2014). Unified polymerization mechanism for the assembly of ASC-dependent inflammasomes. Cell.

[36] Franklin BS, Bossaller L, De Nardo D, Ratter JM, Stutz A, Engels G (2014). The adaptor ASC has extracellular and 'prionoid' activities that propagate inflammation. Nat Immunol.

[37] Fernandes-Alnemri T, Wu J, Yu JW, Datta P, Miller B, Jankowski W (2007). The pyroptosome: a supramolecular assembly of ASC dimers mediating inflammatory cell death via caspase-1 activation. Cell Death Differ.

[38] Masumoto J, Taniguchi S, Ayukawa K, Sarvotham H, Kishino T, Niikawa N (1999). ASC, a novel 22-kDa protein, aggregates during apoptosis of human promyelocytic leukemia HL-60 cells. J Biol Chem.

[39] Hara H, Tsuchiya K, Kawamura I, Fang R, Hernandez-Cuellar E, Shen Y (2013). Phosphorylation of the adaptor ASC acts as a molecular switch that controls the formation of speck-like aggregates and inflammasome activity. Nat Immunol.

[40] Qu Y, Misaghi S, Newton K, Maltzman A, Izrael-Tomasevic A, Arnott D (2016). NLRP3 recruitment by NLRC4 during Salmonella infection. J Exp Med.

[41] Place DE, Kanneganti T-D (2018). Recent advances in inflammasome biology. Curr Opin Immunol.

[42] Martinon F, Mayor A, Tschopp J (2009). The inflammasomes: guardians of the body. Annu Rev Immunol.

[43] Broz P, Dixit VM (2016). Inflammasomes: mechanism of assembly, regulation and signalling. Nat Rev Immunol.

[44] Guo H, Callaway JB, Ting JP (2015). Inflammasomes: mechanism of action, role in disease, and therapeutics. Nat Med.

[45] Jo EK, Kim JK, Shin DM, Sasakawa C (2016). Molecular mechanisms regulating NLRP3 inflammasome activation. Cell Mol Immunol.

[46] Zhao Y, Wang ZC, Feng DC, Zhao HY, Lin MS, Hu Y (2019). p66Shc contributes to liver fibrosis through the regulation of mitochondrial reactive oxygen species. Theranostics.

[47] Zhao YP, Qiu C, Wang WH, Peng JF, Cheng X, Shangguan YT (2020). Cortistatin protects against intervertebral disc degeneration through targeting mitochondrial ROS-dependent NLRP3 inflammasome activation. Theranostics.

[48] Kurundkar D, Kurundkar AR, Bone NB, Becker EJ, Liu WQ, Chacko B (2019). SIRT3 diminishes inflammation and mitigates endotoxin-induced acute lung injury. JCI Insight.

[49] Yi XL, Guo WA, Shi Q, Yang YQ, Zhang WG, Chen XG (2019). SIRT3-dependent mitochondrial dynamics remodeling contributes to oxidative stress-induced melanocyte degeneration in vitiligo. Theranostics.

[50] Fernandes-Alnemri T, Yu JW, Juliana C, Solorzano L, Kang S, Wu JH (2010). The AIM2 inflammasome is critical for innate immunity to Francisella tularensis. Nat Immunol.

[51] Jones JW, Kayagaki N, Broz P, Henry T, Newton K, O'Rourke K (2010). Absent in melanoma 2 is required for innate immune recognition of Francisella tularensis. Proc Natl Acad Sci U S A.

[52] Dai J, Huang YJ, He XH, Zhao M, Wang XZ, Liu ZS (2019). Acetylation blocks cGAS activity and inhibits self-DNA-induced autoimmunity. Cell.

[53] Pi H, Xu S, Reiter RJ, Guo P, Zhang L, Li Y (2015). SIRT3-SOD2-mROS-dependent autophagy in cadmium-induced hepatotoxicity and salvage by melatonin. Autophagy.

[54] Zhao S, Xu W, Jiang W, Yu W, Lin Y, Zhang T (2010). Regulation of cellular metabolism by protein lysine acetylation. Science.

[55] Raghawan AK, Ramaswamy R, Radha V, Swarup G (2019). HSC70 regulates cold-induced caspase-1 hyperactivation by an autoinflammation-causing mutant of cytoplasmic immune receptor NLRC4. Proc Natl Acad Sci U S A.

[56] Fusco WG, Duncan JA (2018). Novel aspects of the assembly and activation of inflammasomes with focus on the NLRC4 inflammasome. Int Immunol.

[57] Duncan JA, Canna SW (2018). The NLRC4 Inflammasome. Immunol Rev.

[58] Luo HZ, Chiang HH, Louw M, Susanto A, Chen D (2017). Nutrient sensing and the oxidative stress response. Trends Endocrinol Metab.

[59] Gao Q, Zhu H (2019). The Overexpression of Sirtuin1 (SIRT1) alleviated lipopolysaccharide (LPS)-induced acute kidney injury (AKI) via inhibiting the activation of nucleotide-binding oligomerization domain-like receptors (NLR) family pyrin domain containing 3 (NLRP3) inflammasome. Med Sci Monit.

[60] Li YX, Yang XF, He YH, Wang WR, Zhang JY, Zhang W (2017). Negative regulation of NLRP3 inflammasome by SIRT1 in vascular endothelial cells. Immunobiology.

[61] Nopparat C, Sinjanakhom P, Govitrapong P (2017). Melatonin reverses H_2_O_2_ -induced senescence in SH-SY5Y cells by enhancing autophagy via sirtuin 1 deacetylation of the RelA/p65 subunit of NF-kappaB. J Pineal Res.

[62] Volt H, Garcia JA, Doerrier C, Diaz-Casado ME, Guerra-Librero A, Lopez LC (2016). Same molecule but different expression: aging and sepsis trigger NLRP3 inflammasome activation, a target of melatonin. J Pineal Res.

[63] Misawa T, Takahama M, Kozaki T, Lee H, Zou J, Saitoh T (2013). Microtubule-driven spatial arrangement of mitochondria promotes activation of the NLRP3 inflammasome. Nat Immunol.

[64] Yu W, Dittenhafer-Reed KE, Denu JM (2012). SIRT3 protein deacetylates isocitrate dehydrogenase 2 (IDH2) and regulates mitochondrial redox status. J Biol Chem.

[65] Liu PH, Xie QH, Wei T, Chen YC, Chen H, Shen WL (2015). Activation of the NLRP3 inflammasome induces vascular dysfunction in obese OLETF rats. Biochem Biophys Res Commun.

[66] Martinon F, Petrilli V, Mayor A, Tardivel A, Tschopp J (2006). Gout-associated uric acid crystals activate the NALP3 inflammasome. Nature.

[67] Mariathasan S, Weiss DS, Newton K, McBride J, O'Rourke K, Roose-Girma M (2006). Cryopyrin activates the inflammasome in response to toxins and ATP. Nature.

[68] Martinon F, Agostini L, Meylan E, Tschopp J (2004). Identification of bacterial muramyl dipeptide as activator of the NALP3/cryopyrin inflammasome. Curr Biol.

[69] Crump JA, Sjolund-Karlsson M, Gordon MA, Parry CM (2015). Epidemiology, clinical presentation, laboratory diagnosis, antimicrobial resistance, and antimicrobial management of invasive Salmonella infections. Clin Microbiol Rev.

[70] Reddy EA, Shaw AV, Crump JA (2010). Community-acquired bloodstream infections in Africa: a systematic review and meta-analysis. Lancet Infect Dis.

