# Supplementary material for: SIRT3-mediated deacetylation of NLRC4 promotes inflammasome activation
Source: Theranostics. 2021 Feb 15;11(8):3981–95. doi: 10.7150/thno.55573 (PMC7914345; doi:10.7150/thno.55573)
Supplement: Supplementary file 1 — Supplementary figure S1. [file thnov11p3981s1.pdf]

Figure S1: SIRT3 is dispensable for NLRP3 and AIM2 inflammasome activation

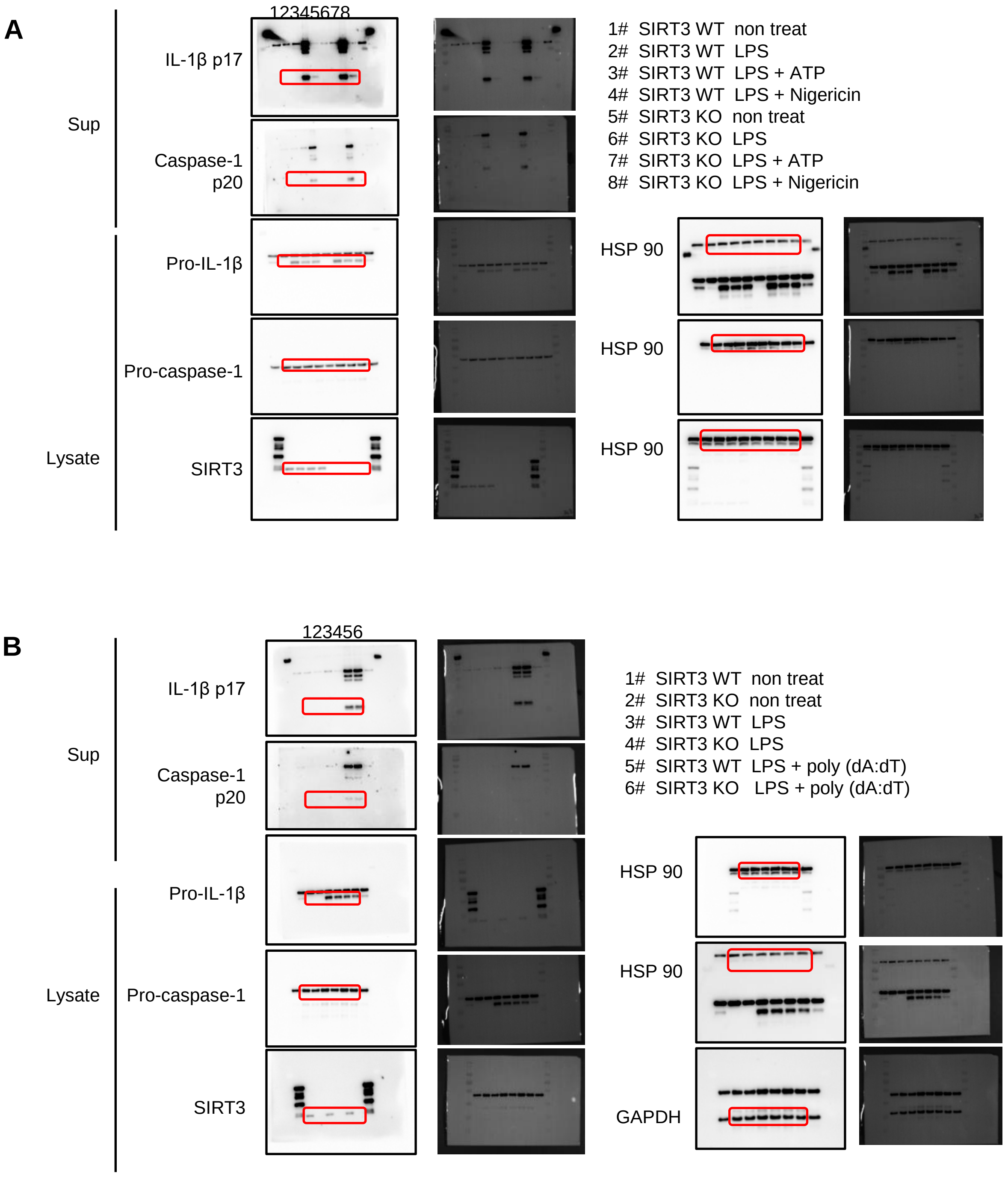

Figure S1: SIRT3 is dispensable for NLRP3 and AIM2 inflammasome activation. (A) WT and SIRT3-deficient peritoneal macrophages were primed with 500 ng/mL LPS for 4 h and then treated with ATP (5 mM) or nigericin (5 μM) for 1 h. Cell lysates and culture supernatants were collected and immunoblotted with the indicated antibodies. (B) WT and SIRT3-deficient peritoneal macrophages were primed with 500ng/mL LPS for 4 h and then transfected with poly(dAdT) (2 μg/mL) for 6 h. Cell lysates and culture supernatants were collected and immunoblotted with the indicated antibodies. Data information: Data are representative of three independent experiments.
